# Thompson Sampling for Non-Stationary Bandit Problems

**DOI:** 10.3390/e27010051

**Published:** 2025-01-09

**Authors:** Han Qi, Fei Guo, Li Zhu

**Affiliations:** School of Software Engineering, Xi’an Jiaotong University, Xi’an 710049, China; co.fly@stu.xjtu.edu.cn

**Keywords:** multi-armed bandits, Thompson sampling, non-stationary

## Abstract

Non-stationary multi-armed bandit (MAB) problems have recently attracted extensive attention. We focus on the abruptly changing scenario where reward distributions remain constant for a certain period and change at unknown time steps. Although Thompson sampling (TS) has shown success in non-stationary settings, there is currently no regret bound analysis for TS with uninformative priors. To address this, we propose two algorithms, discounted TS and sliding-window TS, designed for sub-Gaussian reward distributions. For these algorithms, we establish an upper bound for the expected regret by bounding the expected number of times a suboptimal arm is played. We show that the regret upper bounds of both algorithms are $\tilde{O}\left(\sqrt{TB_{T}}\right)$, where *T* is the time horizon and $B_{T}$ is the number of breakpoints. This upper bound matches the lower bound for abruptly changing problems up to a logarithmic factor. Empirical comparisons with other non-stationary bandit algorithms highlight the competitive performance of our proposed methods.

## 1. Introduction

MAB is a classic sequential decision problem. At each time step, the learner selects an arm from a finite set of arms (also known as actions) based on its past observations, and it only observes the reward of the chosen action. The learner’s goal is to maximize its expected cumulative reward or minimize the regret incurred during the learning process. The regret is defined as the difference between the expected reward of the optimal arm and the expected reward achieved by the MAB algorithm.

MAB has found practical use in various scenarios, with one of the earliest applications being the diagnosis and treatment experiments proposed by Robbins [1]. In this experiment, each patient’s treatment plan corresponds to an arm in the MAB problem, and the goal is to minimize the patient’s health loss by making optimal treatment decisions. Recently, MAB has gained wide-ranging applicability. For example, MAB algorithms have been used in online recommendation systems to improve user experiences and increase engagement [2,3,4]. Similarly, MAB has been employed in online advertising campaigns to optimize the allocation of resources and maximize the effectiveness of ad placements [5]. While the standard MAB model assumes fixed reward distributions, real-world scenarios often involve changing distributions over time. For instance, in online recommendation systems, the collected data gradually become outdated, and user preferences are likely to evolve [6]. This dynamic nature necessitates the development of algorithms that can adapt to these changes, leading to the exploration of non-stationary MAB problems.

In recent years, there has been much research on non-stationary multi-armed bandit problems. These methods can be roughly divided into two categories: they either actively detect changes in the reward distribution using change-point detection algorithms [7,8,9,10,11], or they passively reduce the effect of past observations [12,13,14,15]. Ghatak [16], Alami and Azizi [17] use the active algorithm for non-stationary settings, which combines change-detection and TS. Viappiani [18], Gupta et al. [19], Cavenaghi et al. [20] also address the non-stationary problem with TS algorithm. However, they are experimental paper without theoretical analysis. Liu et al. [21] propose a novel sampling method-predictive sampling. They use information theory tools to analyze the Bayesian regret of their method.

The active methods need to make some assumptions about the change in arms distribution to ensure the effectiveness of the change-point detection algorithm. For instance, refs. [7,8] require a lower bound on the amplitude of change in each arm’s expected rewards. The passive method requires fewer assumptions about the characteristics of the change. They often use a sliding window or discount factor to forget past information to adapt to the change in arms distribution.

However, TS with a passive method has received little theoretical analysis of regret in non-stationary MAB problems. Raj and Kalyani [13] have studied the discounted Thompson sampling with Beta priors. While they only derive the probability of picking a suboptimal arm for the simple case of a two-armed bandit. To the best of our knowledge, only sliding-window Thompson sampling with Beta priors [14] provides the regret upper bounds. However, their proof is not correct. Recently, Fiandri et al. [22] have corrected their proof errors by using the techniques proposed in [23].

Our contributions are as follows: we propose discounted TS (DS-TS) and sliding-window TS (SW-TS) with uninformative priors for abruptly changing settings. We adopt a unified method to analyze the regret upper bound for both algorithms. The theoretical analysis results show that their regret upper bounds are of order $\tilde{O}\left(\sqrt{TB_{T}}\right)$, where *T* is the number of time steps, $B_{T}$ is the number of breakpoints. This regret bound matches the $\Omega (\sqrt{T})$ lower bound proven by Garivier and Moulines [12] in an order sense. We also verify the algorithms in various environmental settings with Gaussian and Bernoulli rewards, and both DS-TS and SW-TS achieve competitive performance.

## 2. Related Works

Many works are based on the idea of forgetting past observations. Discounted UCB (DS-UCB) [12,24] uses a discounted factor to average the past rewards. In order to achieve the purpose of forgetting information, the weight of the early reward is smaller. Garivier and Moulines [12] also propose the sliding-window UCB (SW-UCB) by only using a few recent rewards to compute the UCB index. They calculate the regret upper bound for DS-UCB and SW-UCB as $\tilde{O}\left(\sqrt{TB_{T}}\right)$. EXP3.S, as proposed in [25], has been shown to achieve the regret upper bound by $\tilde{O}\left(\sqrt{TB_{T}}\right)$. Under the assumption that the total variation of the expected rewards over the time horizon is bounded by a budget $V_{T}$, Besbes et al. [26] introduce REXP3 with regret $\tilde{O}\left(T^{2/3}\right)$. Combes and Proutiere [27] propose the SW-OSUB algorithm, specifically for the case of smoothly changing with an upper bound of $\tilde{O}\left(\sigma^{1/4}T\right)$, where σ is the Lipschitz constant of the evolve process. Raj and Kalyani [13] propose the discounted Thompson sampling for Bernoulli priors without providing the regret upper bound. They only calculate the probability of picking a sub-optimal arm for the simple case of a two-armed bandit. Trovo et al. [14] propose the sliding-window Thompson sampling algorithm with regret $\tilde{O}\left(T^{\frac{1+\alpha }{2}}\right)$ for abruptly changing settings and $\tilde{O}\left(T^{\beta }\right)$ for smoothly changing settings. Baudry et al. [15] propose a novel algorithm named Sliding-Window Last Block Subsampling Duelling Algorithm (SW-LB-SDA) with regret $\tilde{O}\left(\sqrt{TB_{T}}\right)$. They assume that the reward distributions belong to the same one-parameter exponential family for all arms during each stationary phase. This means that SW-LB-SDA is not applicable to Gaussian reward distributions with unknown variance.

There are also many works that exploit techniques from the field of change detection to deal with reward distributions varying over time. Mellor and Shapiro [28] combine a Bayesian change point mechanism and Thompson sampling strategy to tackle the non-stationary problem. Their algorithm can detect global switching and per-arm switching. Liu et al. [7] propose a change-detection framework that combines UCB and a change-detection algorithm named CUSUM. They obtain an upper bound for the average detection delay and a lower bound for the average time between false alarms. Cao et al. [8] propose M-UCB, which is similar to CUSUM but uses another simpler change-detection algorithm. M-UCB and CUSUM are nearly optimal, their regret bounds are $\tilde{O}\left(\sqrt{TB_{T}}\right)$.

The above works assume that the rewards distribution are bounded except for SW-LB-SDA. We assume the rewards distribution is a subGaussian distribution, which is a more general setting that includes both bounded distributions and Gaussian distributions.

Recently, there are some works that derive regret bounds without knowing the number of changes. For example, Auer et al. [9] propose an algorithm called ADSWITCH with optimal regret bound $\tilde{O}\left(\sqrt{B_{T}T}\right)$. Suk and Kpotufe [29] improve the work [9] so that the obtained regret bound is smaller than $\tilde{O}\left(\sqrt{ST}\right)$, where *S* only counts the best arms switches. There are also some studies investigating non-stationary representation learning in bandit problems [30,31]. Their focus is mainly on sequential representation learning and introducing an online algorithm that is able to detect task switches and learn and transfer a non-stationary representation in an adaptive fashion.

## 3. Problem Formulation

Assume that the non-stationary MAB problem has *K* arms A:={1,2,…,K} with finite time horizon *T*. At each round *t*, the learner must select an arm $i_{t}\in A$ and obtain the corresponding reward $X_{t}\left(i_{t}\right)$. The rewards are generated from σ-subGaussian distributions. The expectation of $X_{t}\left(i\right)$ is denoted as $\mu_{t}\left(i\right)=E\left[X_{t}\left(i\right)\right]$. A policy π is a function that selects arm $i_{t}$ to play at round *t*. Let $\mu_{t}\left(*\right):=\max_{i\in \{1,\ldots ,K\}}\mu_{t}\left(i\right)$ denote the expected reward of the optimal arm $i_{t}^{*}$ at round *t*. Unlike the stationary MAB settings, where an arm is optimal all of the time (i.e., $\forall t\in \left\{1,\ldots ,T\right\},i_{t}^{*}=i^{*}$), while in the non-stationary settings, the optimal arms might change over time. The performance of a policy π is measured in terms of cumulative expected regret:

(1) $$R_{T}^{\pi }=E\left[\sum_{t=1}^{T}\left(\mu_{t}\left(*\right)-\mu_{t}\left(i_{t}\right)\right)\right],$$

where E[·] is the expectation with respect to randomness of π. Let $\Delta_{t}\left(i\right)=\mu_{t}\left(*\right)-\mu_{t}\left(i\right)$ and let

$$k_{T}\left(i\right)=\sum_{t=1}^{T}1\left\{i_{t}=i,i\neq i_{t}^{*}\right\}$$

denote the number of plays of arm *i* when it is not the best arm until time *T*. When we analyze the upper bound of $R_{T}^{\pi }$, we can directly analyze $E[k_{T}\left(i\right)]$ to obtain the regret upper bound of each arm.

### Abruptly Changing Setting

The abruptly changing setting is introduced by Garivier and Moulines [12] for the first time. The number of breakpoints is denoted as $B_{T}=\sum_{t=1}^{T-1}1\left\{\exists i\in A:\mu_{t}\left(i\right)\neq \mu_{t+1}\left(i\right)\right\}$. Suppose the set of breakpoints is $B=\{b_{1},\ldots ,b_{B_{T}}\}$ (we define $b_{1}=1$). At each breakpoint, the reward distribution changes for at least one arm. The rounds between two adjacent breakpoints are called stationary phase. Abruptly changing bandits pose a more challenging problem, as the learner needs to balance exploration and exploitation within each stationary phase and during the changes between different phases. Trovo et al. [14] makes an assumption about the number of breakpoints to facilitate more generalized analysis, while we explicitly use $B_{T}$ to represent the number of breakpoints for analysis. An implicit assumption we use is that the number of breakpoints $B_{T}$ is much smaller than *T*, i.e., $B_{T}\ll T$. In the community of piecewise stationary bandit problems, it is commonly assumed that $B_{T}$ is much smaller than *T*. When $B_{T}$ and *T* are comparable, researchers typically consider scenarios with smooth changes [27].

## 4. Algorithms

In this section, we propose the DS-TS and SW-TS with uninformative priors for the non-stationary stochastic MAB problems. Different from [32], we assume that the reward distribution follows a σ-subGaussian distribution rather than a bounded distribution. An uninformative prior can be obtained by letting the variance of a Gaussian prior approach infinity. First, assume that $X_{1},\ldots ,X_{n}$ are independently and identically distributed, following a σ-subGaussian distribution with mean μ and the prior distribution is a Gaussian distribution $N(0,\sigma_{0}^{2})$. The posterior distribution is also a Gaussian distribution $N(\mu_{1},\sigma_{1}^{2})$ where

$$\mu_{1}=\sigma_{1}^{2}\left(\frac{0}{\sigma_{0}^{2}}+\frac{\sum_{i=1}^{n}X_{i}}{\sigma^{2}}\right),\sigma_{1}^{2}=\frac{1}{\frac{1}{\sigma_{0}^{2}}+\frac{n}{\sigma^{2}}}.$$

Let $\sigma_{0}=+\infty$, we obtain the posterior distribution as $N(\frac{1}{n}\sum_{i=1}^{n}X_{i},\frac{\sigma^{2}}{n})$. In fact, when $\sigma_{0}$ is infinite, the prior distribution turns to be an uninformative prior.

### 4.1. DS-TS

DS-TS uses a discount factor γ (0<γ<1) to dynamically adjust the estimate of each arm’s distribution. The key to our algorithm is to decrease the sampling variance of the selected arm while increasing the sampling variance of the unselected arms.

Specifically, let

$$N_{t}\left(\gamma ,i\right)=\sum_{j=1}^{t}\gamma^{t-j}1\left\{i_{j}=i\right\}$$

denote the discounted number of plays of arm *i* until time *t*. We use

$${\hat{\mu }}_{t}\left(\gamma ,i\right)=\frac{1}{N_{t}\left(\gamma ,i\right)}\sum_{j=1}^{t}\gamma^{t-j}X_{j}\left(i\right)1\left\{i_{j}=i\right\}$$

called discounted empirical average to estimate the expected rewards of arm *i*. In non-stationary settings, we use the discounted average and discounted number of plays instead of the true average and number of plays, respectively. Therefore, the posterior distribution is $N({\hat{\mu }}_{t}\left(\gamma ,i\right),\frac{\sigma^{2}}{N_{t}\left(\gamma ,i\right)})$.

Algorithm 1 shows the pseudocode of DS-TS. Step 3 is the Thompson sampling. For each arm, we draw a random sample $\theta_{t}\left(i\right)$ from $N({\hat{\mu }}_{t}\left(\gamma ,i\right),\frac{4\sigma^{2}}{N_{t}\left(\gamma ,i\right)})$. We use $\frac{4\sigma^{2}}{N_{t}\left(\gamma ,i\right)}$ as the posterior variance instead of $\frac{\sigma^{2}}{N_{t}\left(\gamma ,i\right)}$, which helps the subsequent analysis. Then, we select arm $i_{t}$ with the maximum sample value and obtain the reward $X_{t}\left(i_{t}\right)$ (Step 5). To avoid the time complexity going to $O(T^{2})$, we introduce ${\tilde{\mu }}_{t}\left(\gamma ,i\right)=\sum_{j=1}^{t}\gamma^{t-j}X_{j}\left(i\right)1\left\{i_{j}=i\right\}$ to calculate ${\hat{\mu }}_{t}\left(\gamma ,i\right)$ using an iterative method (Steps 7–9).

If arm *i* is selected at round *t*, the posterior distribution is updated as follows:

$${\hat{\mu }}_{t+1}\left(\gamma ,i\right)=\frac{\gamma {\hat{\mu }}_{t}\left(\gamma ,i\right)N_{t}\left(\gamma ,i\right)+X_{t}\left(i\right)}{\gamma N_{t}\left(\gamma ,i\right)+1}=\frac{{\tilde{\mu }}_{t+1}\left(\gamma ,i\right)}{N_{t+1}\left(\gamma ,i\right)}$$

If arm *i* is not selected at round *t*, the posterior distribution is updated as

$${\hat{\mu }}_{t+1}\left(\gamma ,i\right)=\frac{{\tilde{\mu }}_{t+1}\left(\gamma ,i\right)}{N_{t+1}\left(\gamma ,i\right)}=\frac{\gamma {\tilde{\mu }}_{t}\left(\gamma ,i\right)}{\gamma N_{t}\left(\gamma ,i\right)}={\hat{\mu }}_{t}\left(\gamma ,i\right)$$

i.e., the expectation of posterior distribution remains unchanged.
**Algorithm 1** DS-TS 1:discounted factor γ∈(0,1), ${\hat{\mu }}_{1}\left(i\right)=0$, ${\tilde{\mu }}_{1}\left(i\right)=0$, $N_{t}\left(\gamma ,i\right)=0$ 2:**for** t=1,…,T **do** 3:   **for** i=1,…,K **do** 4:     sample $\theta_{t}\left(i\right)∼N\left({\hat{\mu }}_{t}\left(\gamma ,i\right),\frac{4\sigma^{2}}{N_{t}\left(\gamma ,i\right)}\right)$ 5:   **end for** 6:   Pull arm $i_{t}=\arg\max_{i}\theta_{t}\left(i\right)$, observe reward $X_{t}\left(i_{t}\right)$ 7:   **for** i=1,…,K **do** 8:     ${\tilde{\mu }}_{t+1}\left(\gamma ,i\right)=\gamma {\tilde{\mu }}_{t}\left(\gamma ,i\right)+1\left\{i_{t}=i\right\}X_{t}\left(i\right)$ 9:     $N_{t+1}\left(\gamma ,i\right)=\gamma N_{t}\left(\gamma ,i\right)+1\left\{i_{t}=i\right\}$10:     ${\hat{\mu }}_{t+1}\left(\gamma ,i\right)=\frac{{\tilde{\mu }}_{t+1}\left(\gamma ,i\right)}{N_{t+1}\left(\gamma ,i\right)}$11:   **end for**12:**end for**

### 4.2. SW-TS

SW-TS uses a sliding window τ to adapt to changes in the reward distribution. Let

$$N_{t}\left(\tau ,i\right)=\sum_{j=t-\tau +1}^{t}1\left\{i_{j}=i\right\},{\hat{\mu }}_{t}\left(\tau ,i\right)=\frac{1}{N_{t}\left(\tau ,i\right)}\sum_{j=t-\tau +1}^{t}X_{j}\left(i\right)1\left\{i_{j}=i\right\}.$$

If t<τ, the range of summation is from 1 to *t*. Similar to DS-TS, the posterior distribution is $N({\hat{\mu }}_{t}\left(\tau ,i\right),\frac{4\sigma^{2}}{N_{t}\left(\tau ,i\right)})$. Algorithm 2 shows the pseudocode of SW-TS. To avoid the time complexity going to $O(T^{2})$, we introduce ${\tilde{\mu }}_{t}\left(\tau ,i\right)=\sum_{j=t-\tau +1}^{t}X_{j}\left(i\right)1\left\{i_{j}=i\right\}$ to update ${\hat{\mu }}_{t}\left(\tau ,i\right)$.
**Algorithm 2** SW-TS 1:sliding window τ, ${\hat{\mu }}_{1}\left(i\right)=0$, ${\tilde{\mu }}_{1}\left(i\right)=0$, $N_{t}\left(\tau ,i\right)=0$ 2:**for** t=1,…,T **do** 3:   **for** i=1,…,K **do** 4:     sample $\theta_{t}\left(i\right)∼N\left({\hat{\mu }}_{t}\left(\tau ,i\right),\frac{4\sigma^{2}}{N_{t}\left(\tau ,i\right)}\right)$ 5:   **end for** 6:   Pull arm $i_{t}=\arg\max_{i}\theta_{t}\left(i\right)$, observe reward $X_{t}\left(i_{t}\right)$ 7:   **for** i=1,…,K **do** 8:     $N_{t+1}\left(\tau ,i\right)=N_{t}\left(\tau ,i\right)+1\left\{i_{t}=i\right\}-1\left\{i_{t-\tau }=i\right\}$ 9:     ${\tilde{\mu }}_{t+1}\left(\tau ,i\right)={\tilde{\mu }}_{t}\left(\tau ,i\right)+1\left\{i_{t}=i\right\}X_{t}\left(i\right)-1\left\{i_{t-\tau }=i\right\}X_{t-\tau }\left(i\right)$10:     ${\hat{\mu }}_{t+1}\left(\tau ,i\right)=\frac{{\tilde{\mu }}_{t+1}\left(\tau ,i\right)}{N_{t+1}\left(\tau ,i\right)}$11:   **end for**12:**end for**

### 4.3. Results

In this section, we give the regret upper bounds of DS-TS and SW-TS. Then, we discuss how to take the values of the parameters so that these algorithms reach the optimal upper bound.

Recall that $\Delta_{t}\left(i\right)=\mu_{t}\left(*\right)-\mu_{t}\left(i\right)$. Let $\Delta_{T}\left(i\right)=\min\left\{\Delta_{t}\left(i\right):t\leq T,i\neq i_{t}^{*}\right\}$, be the minimum difference between the expected reward of the best arm $i_{t}^{*}$ and the expected reward of arm *i* in all time *T* when the arm *i* is not the best arm. Let $\Delta_{max}^{T}=\max\left\{\mu_{t_{1}}\left(i\right)-\mu_{t_{2}}\left(i\right):t_{1}\neq t_{2},i\in \left[K\right]\right\}$ denote the maximum expected variation of arms.

**Theorem** **1** (DS-TS). *Let γ∈(0,1) satisfying ${\left(\frac{\sigma }{\Delta_{max}^{T}}\right)}^{2}<\frac{e}{1-\gamma }$. For any suboptimal arm i,*

$$E\left[k_{T}\left(i\right)\right]\leq B_{T}D\left(\gamma \right)+C_{1}\left(\gamma \right)L_{1}\left(\gamma \right)\gamma^{-\frac{1}{1-\gamma }}T\left(1-\gamma \right),$$

*where*

$$D\left(\gamma \right)=\frac{\log({\left(\frac{\sigma }{\Delta_{max}^{T}}\right)}^{2}{\left(1-\gamma \right)}^{2}\log\frac{1}{1-\gamma })}{\log\gamma },C_{1}\left(\gamma \right)=e^{17}+15\log\frac{1}{1-\gamma },$$

$$L_{1}\left(\gamma \right)=\frac{1152\log\left(\frac{1}{1-\gamma }+e^{17}\right)\sigma^{2}}{\gamma^{1/(1-\gamma )}{\left(\Delta_{T}\left(i\right)\right)}^{2}}.$$

**Remark** **1.** *The condition ${\left(\frac{\sigma }{\Delta_{max}^{T}}\right)}^{2}<\frac{e}{1-\gamma }$ can ensure that D(γ) is well defined. In general, we do not need to know $\Delta_{max}^{T}$ in advance when setting the value of γ. If we choose a γ close to 1, then the condition ${\left(\frac{\sigma }{\Delta_{max}^{T}}\right)}^{2}<\frac{e}{1-\gamma }$ in Theorem 1 is easily satisfied, as shown in the corollary below.*

**Corollary** **1.** *If the time horizon T and number of breakpoints $B_{T}$ are known in advance, the discounted factor can be chosen as $\gamma =1-\frac{1}{\sigma }\sqrt{\frac{B_{T}}{T\log T}}$. If $B_{T}\ll T$,*

$${\left(\frac{\sigma }{\Delta_{max}^{T}}\right)}^{2}\left(1-\gamma \right)=\frac{\sigma }{{\left(\Delta_{max}^{T}\right)}^{2}}\sqrt{\frac{B_{T}}{T\log T}}<e.$$

*we have*

$$E\left[k_{T}\left(i\right)\right]=O\left(\sqrt{TB_{T}}{\left(\log T\right)}^{\frac{3}{2}}\right).$$

**Theorem** **2** (SW-TS). *Let τ>0, for any suboptimal arm i,*

$$E\left[k_{T}\left(i\right)\right]\leq B_{T}\tau +C_{2}\left(\tau \right)L_{2}\left(\tau \right)\frac{T}{\tau },$$

*where*

$$C_{2}\left(\tau \right)=e^{11}+15\log\tau ,L_{2}\left(\tau \right)=\frac{1152\log\left(\tau +e^{11}\right)\sigma^{2}}{{\left(\Delta_{T}\left(i\right)\right)}^{2}}.$$

**Corollary** **2.** *If the time horizon T and number of breakpoints $B_{T}$ are known in advance, the sliding window can be chosen as $\tau =\sigma \sqrt{T/B_{T}}\log T$, then*

$$E\left[k_{T}\left(i\right)\right]=O\left(\sqrt{TB_{T}}\log T\right).$$

## 5. Proofs of Upper Bounds

Before giving the detailed proof, we discuss the main challenges in regret analysis of Thompson sampling in a non-stationary setting. These challenges are addressed by Lemmas 1–3.

### 5.1. Challenges in Regret Analysis

Existing analyses of regret bounds for Thompson sampling [32,33,34] decompose the regret into two parts. The first part of regret comes from the over-estimation of the suboptimal arm, which can be dealt with by the concentration properties of the sampling distribution and rewards distribution. The second part is the under-estimation of the optimal arm, which mainly relies on bounding the following equation.

(2) $$\sum_{t=1}^{T}E\left[\frac{1-p_{i,t}}{p_{i,t}}1\left\{i_{t}=i_{t}^{*},\theta_{t}\left(*\right)\leq \mu_{t}\left(*\right)-\epsilon_{i}\right\}\right],$$

where $p_{i,t}=P\left(\theta_{t}\left(*\right)>\mu_{t}\left(*\right)-\epsilon_{i}\right)$ is the probability that the best arm will not be under-estimated from the mean reward by a margin $\epsilon_{i}$.

The first challenge is specific to the DS-TS algorithm. Unlike SW-TS, which completely forgets previous information after τ rounds following a breakpoint, DS-TS cannot fully forget past information.

This makes it challenging to utilize the concentration properties of the reward distribution to bound regret comes from the over-estimate of the suboptimal arm. And this will further affect the analysis of Equation (2).

The second challenge is the under-estimation of the optimal arm. In stationary settings, $p_{i,t}$ changes only when the optimal arm is selected, Equation (2) can be bounded by the method proposed by Agrawal and Goyal [32]. However, the distribution of $\theta_{t}\left(*\right)$ may vary over time in non-stationary settings. It is challenging and nontrivial to obtain a tight bound of Equation (2).

To overcome the first challenges, we adjust the posterior variance to be $\frac{4\sigma^{2}}{N_{t}\left(\gamma ,i\right)}$. This slightly larger variance is specifically designed for the $\sigma^{2}$-subGaussian distribution, which helps to bound $E[\frac{1}{p_{i,t}}]$ (In Appendix B.4, we have shown that our analysis method requires the variance to be greater than $\frac{2\sigma^{2}}{N}$. And we set the variance to $\frac{4\sigma^{2}}{N}$ for a more convenient presentation of the paper’s results.). Then, we define $U_{t}\left(\gamma ,i\right)$, which serves a role similar to the upper confidence bound in the UCB algorithm. We solve this problem through Lemmas 1 and 2.

For the second challenge, we use the new defined $U_{t}\left(\gamma ,i\right)$ and employ a new regret decomposition for Equation (2) based on whether the event $\left\{N_{t}\left(\gamma ,*\right)>L_{1}\left(\gamma \right)\right\}$ occurs. Intuitively, if $N_{t}\left(\gamma ,*\right)>L_{1}\left(\gamma \right)$, $p_{i,t}$ is close to 1, which will lead to a sharp bound. If $N_{t}\left(\gamma ,*\right)\leq L_{1}\left(\gamma \right)$, using Lemma A3 we can also obtain the upper bound of Equation (2). We derive the upper bound of $E[\frac{1}{p_{i,t}}]$ for non-stationary settings, with an extra logarithmic term compared with the stationary settings. The proof of Lemma 3 in Appendix B.3 demonstrates these details.

### 5.2. Proofs of Theorem 1

For arm $i\neq i_{t}^{*}$, we choose two threshold $x_{t}\left(i\right),y_{t}\left(i\right)$ such that $x_{t}\left(i\right)=\mu_{t}\left(i\right)+\frac{\Delta_{t}\left(i\right)}{3},y_{t}\left(i\right)=\mu_{t}\left(*\right)-\frac{\Delta_{t}\left(i\right)}{3}$. Then $\mu_{t}\left(i\right)<x_{t}\left(i\right)<y_{t}\left(i\right)<\mu_{t}\left(*\right)$ and $y_{t}\left(i\right)-x_{t}\left(i\right)=\frac{\Delta_{t}\left(i\right)}{3}$. The history $F_{t}$ is defined as the plays and rewards of the previous *t* plays. ${\hat{\mu }}_{t}\left(\gamma ,i\right),i_{t}$ and the distribution of $\theta_{t}\left(i\right)$ are determined by the history $F_{t-1}$.

The abruptly changing setting is in fact piecewise-stationary. The rounds between two adjacent breakpoints are stationary. Based on this observation, we define the pseudo-stationary phase as

$$T\left(\gamma \right)=\{t\leq T:\forall s\in \left(t-D\left(\gamma \right),t\right],\mu_{s}\left(\cdot \right)=\mu_{t}\left(\cdot \right)\}.$$

The rounds in T(γ) can to some extent ensure that the rewards are “stationary”. For any t∈T(γ), the rewards distribution remain unchanged between (t−D(γ),t). Therefore, we can obtain a good estimate of the rewards distribution in T(γ) (Lemma 1). Let S(γ) denote the complement of T(γ), i.e., S(γ)={t≤T:t∉T(γ)}. Note that, there is at most D(γ) rounds belonging to S(γ) after each breakpoint. This is because the rounds between two adjacent breakpoints are stationary. The time steps after the breakpoint D(γ) rounds, the rewards distribution do not change and therefore belongs to T(γ). Therefore, the number of elements in the set S(γ) has an upper bound $B_{T}D\left(\gamma \right)$, i.e.,

(3) $$\left|S\left(\gamma \right)\right|\leq B_{T}D\left(\gamma \right)$$

**Figure 1:**
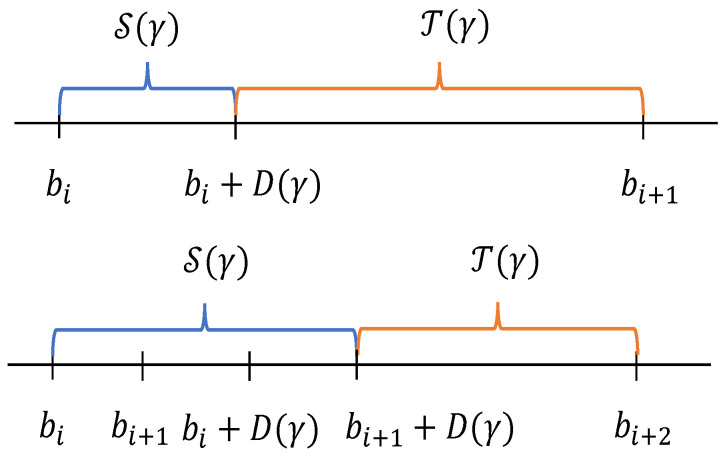
Illustration of T(γ) and S(γ) in two different situations. $b_{i},b_{i+1},b_{i+2}$ are the breakpoints. The situation that $b_{i+1}-b_{i}>D\left(\gamma \right)$ is shown in the top figure, and $b_{i+1}-b_{i}\leq D\left(\gamma \right)$ is in the bottom.

Figure 1 shows T(γ) and S(γ) in two different situations. Since during the rounds in S(γ), i.e., the rounds following a breakpoint, the estimate of the expected rewards may be poor, we directly bound the regret during S(γ) by $B_{T}D\left(\gamma \right)$ and only focus on the regret in T(γ).

To facilitate the analysis, we define the following quantities and events.

(4) $$n=6\sqrt{2}+3\sqrt{1-\gamma },A\left(\gamma \right)=\frac{n^{2}\log\left(\frac{1}{1-\gamma }\right)\sigma^{2}}{{\left(\Delta_{T}\left(i\right)\right)}^{2}},U_{t}\left(\gamma ,i\right)=\sigma \sqrt{\frac{\left(1-\gamma \right)\log\frac{1}{1-\gamma }}{N_{t}\left(\gamma ,i\right)}}.$$

**Definition** **1.** *Define $E_{t}\left(\gamma ,i\right)$ as the event $\left\{i_{t}=i,N_{t}\left(\gamma ,i\right)>A\left(\gamma \right)\right\}$. Define $E_{t}^{\theta }\left(i\right)$ as the event $\left\{\theta_{t}\left(i\right)<y_{t}\left(i\right)\right\}$.*

Intuitively, event $E_{t}\left(\gamma ,i\right)$ represents selecting a sufficiently explored suboptimal arm. Event $E_{t}^{\theta }\left(i\right)$ denotes $\theta_{t}\left(i\right)$ is not too far from the mean $\mu_{t}\left(i\right)$.

Now we list some useful lemmas. The detailed proofs are provided in the appendix. The following lemma depicts that after finite rounds at the breakpoint, i.e., in the pseudo-stationary phase, the distance between $\mu_{t}\left(i\right)$ and discounted average of expectation for arm *i* can be bounded by $U_{t}\left(\gamma ,i\right)$. $U_{t}\left(\gamma ,i\right)$ is analogous to the upper confidence bound in the UCB algorithm.

**Lemma** **1.** *Let ${\ddot{\mu }}_{t}\left(\gamma ,i\right)=\frac{1}{N_{t}\left(\gamma ,i\right)}\sum_{j=1}^{t}\gamma^{t-j}1\left\{i_{j}=i\right\}\mu_{j}\left(i\right)$ denote the discounted average of expectation for arm i at time step t. ∀t∈T(γ), the distance between $\mu_{t}\left(i\right)$ and ${\ddot{\mu }}_{t}\left(\gamma ,i\right)$ is less than $U_{t}\left(\gamma ,i\right)$.*

(5) $$|\mu_{t}\left(i\right)-{\ddot{\mu }}_{t}\left(\gamma ,i\right)|\leq U_{t}\left(\gamma ,i\right),$$

Using Lemma 1 and the self-normalized Hoeffding-type inequality for subGaussian distributions (Lemma A1), we have the following lemma. This lemma helps to bound regret comes from the over-estimation of suboptimal arm.

**Lemma** **2.** *$\forall t\in T\left(\gamma \right),i\neq i_{t}^{*}$,*

$$P\left({\hat{\mu }}_{t}\left(\gamma ,i\right)>x_{t}\left(i\right),N_{t}\left(\gamma ,i\right)>A\left(\gamma \right)\right)\leq {\left(1-\gamma \right)}^{2}$$

The following key lemma helps bound the regret comes from the under-estimation of the optimal arm. This is the most tricky part of analyzing TS. Note that, the proof in [14] does not prove the result of the following lemma.

**Lemma** **3.** *Let $p_{i,t}=P\left(\theta_{t}\left(*\right)>y_{t}\left(i\right)|F_{t-1}\right)$. For any t∈T(γ) and $i\neq i_{t}^{*}$,*

$$\begin{array}{c} \sum_{t\in T(\gamma )}E\left[\frac{1-p_{i,t}}{p_{i,t}}1\left\{i_{t}=i_{t}^{*},\theta_{t}\left(i\right)<y_{t}\left(i\right)\right\}\right]\leq \left(e^{17}+12\log\frac{1}{1-\gamma }\right)T\left(1-\gamma \right)L_{1}\left(\gamma \right)\gamma^{-\frac{1}{1-\gamma }}. \end{array}$$

Before we give the detailed proof, we give a outline of our proof.

*Proof Outlines.* First, since the regret incurred in {t∉T(γ)} can be bounded by $B_{T}D\left(\gamma \right)$, we only consider the regret in rounds {t∈T(γ)}. Then, we consider the event $\left\{N_{t}\left(\gamma ,i\right)>A\left(\gamma \right)\right\}$. If this event is not true, we can use Lemma A3 to bound the regret by $T\left(1-\gamma \right)A\left(\gamma \right)\gamma^{-1/(1-\gamma )}$. If this event holds true, we additionally consider whether the suboptimal arm is over-estimation (${\hat{\mu }}_{t}\left(\gamma ,i\right)>x_{t}\left(i\right)$) and whether event $\left\{\theta_{t}\left(i\right)<y_{t}\left(i\right)\right\}$ is true to decompose the regret into three parts as Equation (10). The first part comes from the over-estimation of the suboptimal arm, which can be bounded by Lemma 2. The second part comes from the bias in sampling the suboptimal arm, which can be bounded by the properties of Gaussian distribution Equation (A1). The third part denotes that the regret comes from the under-estimation of the optimal arm and can be bounded by Lemma 3.

The proof is in 5 steps:

**Step 1:** We can divide the rounds t∈{1,…,T} into two parts: {t∈T(γ)} and {t∉T(γ)}. Equation (3) shows that the number of elements in the second part is smaller than $B_{T}D\left(\gamma \right)$, we have

(6) $$E\left[k_{T}\left(i\right)\right]\leq B_{T}D\left(\gamma \right)+\sum_{t\in T(\gamma )}P\left(i_{t}=i\right).$$

**Step 2:** Then, we consider the event $\left\{N_{t}\left(\gamma ,i\right)>A\left(\gamma \right)\right\}$.

$$\begin{array}{c} \sum_{t\in T(\gamma )}P\left(i_{t}=i\right)=\sum_{t\in T(\gamma )}P\left(i_{t}=i,N_{t}\left(\gamma ,i\right)<A\left(\gamma \right)\right)+\sum_{t\in T(\gamma )}P\left(i_{t}=i,N_{t}\left(\gamma ,i\right)>A\left(\gamma \right)\right). \end{array}$$

We first bound $\sum_{t\in T(\gamma )}P\left(i_{t}=i,N_{t}\left(\gamma ,i\right)<A\left(\gamma \right)\right)$.

(7) $$\begin{array}{cc} \sum_{t\in T(\gamma )}P\left(i_{t}=i,N_{t}\left(\gamma ,i\right)<A\left(\gamma \right)\right) & =\sum_{t\in T(\gamma )}E\left[P(i_{t}=i,N_{t}\left(\gamma ,i\right)<A\left(\gamma \right)|F_{t-1})\right]\\ \; & =\sum_{t\in T(\gamma )}E\left[E\left[1\{i_{t}=i,N_{t}\left(\gamma ,i\right)<A\left(\gamma \right)|F_{t-1}\}\right]\right]\\ \; & =\sum_{t\in T(\gamma )}E\left[1\{i_{t}=i,N_{t}\left(\gamma ,i\right)<A\left(\gamma \right)\}\right], \end{array}$$

where the last equation uses the tower rule of expectation.

Using Lemma A3, we have

(8) $$\sum_{t\in T(\gamma )}P\left(i_{t}=i,N_{t}\left(\gamma ,i\right)<A\left(\gamma \right)\right)\leq T\left(1-\gamma \right)A\left(\gamma \right)\gamma^{-1/(1-\gamma )}$$

Therefore,

(9) $$\begin{array}{c} E\left[k_{T}\left(i\right)\right]\leq T\left(1-\gamma \right)A\left(\gamma \right)\gamma^{-\frac{1}{1-\gamma }}+B_{T}D\left(\gamma \right)+\sum_{t\in T(\gamma )}P\left(i_{t}=i,N_{t}\left(\gamma ,i\right)>A\left(\gamma \right)\right) \end{array}$$

**Step 3:** Recall that we use $E_{t}\left(\gamma ,i\right)$ to denote the event $\left\{i_{t}=i,N_{t}\left(\gamma ,i\right)>A\left(\gamma \right)\right\}$ and $E_{t}^{\theta }\left(i\right)$ denote the event $\theta_{t}\left(i\right)<y_{t}\left(i\right)$. Equation (9) may be decomposed as follows:

(10) $$\begin{array}{cc} \sum_{t\in T(\gamma )}P\left(E_{t}\left(\gamma ,i\right)\right) & =\sum_{t\in T(\gamma )}P\left(E_{t}\left(\gamma ,i\right),{\hat{\mu }}_{t}\left(\gamma ,i\right)>x_{t}\left(i\right)\right)\\ \; & \;+\sum_{t\in T(\gamma )}P\left(E_{t}\left(\gamma ,i\right),{\hat{\mu }}_{t}\left(\gamma ,i\right)<x_{t}\left(i\right),\bar{E_{t}^{\theta }\left(i\right)}\right)\\ \; & \;+\sum_{t\in T(\gamma )}P\left(E_{t}\left(\gamma ,i\right),{\hat{\mu }}_{t}\left(\gamma ,i\right)<x_{t}\left(i\right),E_{t}^{\theta }\left(i\right)\right) \end{array}$$

Using Lemma 2, the first part in Equation (10) can be bounded by $T{\left(1-\gamma \right)}^{2}$.

**Step 4:** Then, we bound the second part in Equation (10). Use the fact that $N_{t}\left(\gamma ,i\right)$ and ${\hat{\mu }}_{t}\left(i\right)$ are determined by the history $F_{t-1}$, we have

(11) $$\begin{array}{cc} \; & \sum_{t\in T(\gamma )}P\left(E_{t}\left(\gamma ,i\right),{\hat{\mu }}_{t}\left(\gamma ,i\right)<x_{t}\left(i\right),\bar{E_{t}^{\theta }\left(i\right)}\right)\\ \; & =E\left[\sum_{t\in T(\gamma )}E\left[1\left\{i_{t}=i,N_{t}\left(\gamma ,i\right)>A\left(\gamma \right),{\hat{\mu }}_{t}\left(\gamma ,i\right)<x_{t}\left(i\right),\bar{E_{t}^{\theta }\left(i\right)}\right\}|F_{t-1}\right]\right]\\ \; & =E\left[\sum_{t\in T(\gamma )}1\left\{N_{t}\left(\gamma ,i\right)>A\left(\gamma \right),{\hat{\mu }}_{t}\left(\gamma ,i\right)<x_{t}\left(i\right)\right\}P\left(i_{t}=i,\bar{E_{t}^{\theta }\left(i\right)}|F_{t-1}\right)\right]\\ \; & \leq E\left[\sum_{t\in T(\gamma )}1\left\{N_{t}\left(\gamma ,i\right)>A\left(\gamma \right),{\hat{\mu }}_{t}\left(\gamma ,i\right)<x_{t}\left(i\right)\right\}P\left(\theta_{t}\left(i\right)>y_{t}\left(i\right)|F_{t-1}\right)\right]. \end{array}$$

Given the history $F_{t-1}$ such that $N_{t}\left(\gamma ,i\right)>A\left(\gamma \right)$ and ${\hat{\mu }}_{t}\left(\gamma ,i\right)<x_{t}\left(i\right)$, we have

$$y_{t}\left(i\right)-{\hat{\mu }}_{t}\left(\gamma ,i\right)>y_{t}\left(i\right)-x_{t}\left(i\right)=\frac{\Delta_{t}\left(i\right)}{3}\geq \frac{\Delta_{T}\left(i\right)}{3}.$$

Therefore,

(12) $$\begin{array}{cc} \; & P\left(\theta_{t}\left(i\right)>y_{t}\left(i\right)|F_{t-1}\right))\leq P\left(\theta_{t}\left(i\right)-{\hat{\mu }}_{t}\left(\gamma ,i\right)>\frac{\Delta_{T}\left(i\right)}{3}|F_{t-1}\right)\\ \; & \leq \frac{1}{2}\exp\left(-\frac{{\left(\Delta_{T}\left(i\right)\right)}^{2}A\left(\gamma \right)}{72\sigma^{2}}\right)\\ \; & \leq \frac{1}{2}\left(1-\gamma \right), \end{array}$$

where the second inequality follows $\theta_{t}\left(i\right)∼N\left({\hat{\mu }}_{t}\left(\gamma ,i\right),\frac{4\sigma^{2}}{N_{t}\left(\gamma ,i\right)}\right)$ and Equation (A1).

For other $F_{t-1}$, the indicator term $1\{N_{t}\left(\gamma ,i\right)>A\left(\gamma \right),{\hat{\mu }}_{t}\left(\gamma ,i\right)<x_{t}\left(i\right)\}$ will be 0. Hence, we can bound the second part by $\frac{T}{2}\left(1-\gamma \right)$

**Step 5:** Finally, we focus the third term in Equation (10). Using Lemma A2 and the fact that $p_{i,t}$ is fixed given $F_{t-1}$,

$$\begin{array}{cc} \sum_{t\in T(\gamma )}P\left(E_{t}\left(\gamma ,i\right),{\hat{\mu }}_{t}\left(\gamma ,i\right)<x_{t}\left(i\right),E_{t}^{\theta }\left(i\right)\right) & \leq \sum_{t\in T(\gamma )}E\left[\frac{1-p_{i,t}}{p_{i,t}}P\left(i_{t}=i_{t}^{*},E_{t}^{\theta }\left(i\right)|F_{t-1}\right)\right]\\ & =\sum_{t\in T(\gamma )}E\left[E\left[\frac{1-p_{i,t}}{p_{i,t}}1\left\{i_{t}=i_{t}^{*},E_{t}^{\theta }\left(i\right)|F_{t-1}\right\}\right]\right]\\ & =\sum_{t\in T(\gamma )}E\left[\frac{1-p_{i,t}}{p_{i,t}}1\left\{i_{t}=i_{t}^{*},E_{t}^{\theta }\left(i\right)\right\}\right] \end{array}$$

Then, by Lemma 3, we have

(13) $$\sum_{t\in T(\gamma )}P\left(E_{t}\left(\gamma ,i\right),{\hat{\mu }}_{t}\left(\gamma ,i\right)<x_{t}\left(i\right),E_{t}^{\theta }\left(i\right)\right)\leq \left(e^{17}+12\log\frac{1}{1-\gamma }\right)T\left(1-\gamma \right)L_{1}\left(\gamma \right)\gamma^{-\frac{1}{1-\gamma }}.$$

Substituting the results in Step 3–5 to Equation (10) and Equation (9),

$$\begin{array}{cc} E[k_{T}\left(i\right)] & \leq T\left(1-\gamma \right)A\left(\gamma \right)\gamma^{-1/(1-\gamma )}+B_{T}D\left(\gamma \right)+2T\left(1-\gamma \right)\\ & \;+\left(e^{17}+12\log\frac{1}{1-\gamma }\right)T\left(1-\gamma \right)L_{1}\left(\gamma \right)\gamma^{-1/(1-\gamma )}\\ & \leq B_{T}D\left(\gamma \right)+\left(e^{17}+15\log\frac{1}{1-\gamma }\right)L_{1}\left(\gamma \right)\gamma^{-\frac{1}{1-\gamma }}T\left(1-\gamma \right). \end{array}$$

### 5.3. Proofs of Theorem 2

The proof of Theorem 2 is similar to Theorem 1. The main difference is that the pseudo-stationary phase is now defined as $T\left(\tau \right)=\{t\leq T:\forall s\in \left(t-\tau ,t\right],\mu_{s}\left(\cdot \right)=\mu_{t}\left(\cdot \right)\}$. Let

$${\ddot{\mu }}_{t}\left(\tau ,i\right)=\frac{1}{N_{t}\left(\tau ,i\right)}\sum_{j=t-\tau +1}^{t}1\left\{i_{j}=i\right\}\mu_{j}\left(i\right).$$

If t∈T(τ),

$${\ddot{\mu }}_{t}\left(\tau ,i\right)=\frac{1}{N_{t}\left(\tau ,i\right)}\sum_{j=t-\tau +1}^{t}1\left\{i_{j}=i\right\}\mu_{t}\left(i\right)=\mu_{t}\left(i\right).$$

This means the bias ($U_{t}\left(\gamma ,i\right)$) vanishes. We no longer need an *n* related to τ to deal with the bias issue. We only need to define A(τ) as

$$A\left(\tau \right)=\frac{72\log\left(\tau \right)\sigma^{2}}{{\left(\Delta_{T}\left(i\right)\right)}^{2}}.$$

We directly list the following two lemmas, corresponding to Lemma 2 and Lemma 3, respectively.

**Lemma** **4.** *$\forall t\in T\left(\tau \right),t\neq i_{t}^{*}$,*

$$P\left({\hat{\mu }}_{t}\left(\tau ,i\right)>x_{t}\left(i\right),N_{t}\left(\tau ,i\right)>A\left(\tau \right)\right)\leq \frac{1}{\tau^{2}}.$$

This lemma is similar to Lemma 2. It can be used to bound the regret that comes from over-estimation of the suboptimal arms. This lemma can be proved by Hoeffding-type inequality for subGaussian distributions (Lemma A1). The detailed proofs can be found in Appendix B.5.

**Lemma** **5.** *Let $p_{i,t}=P\left(\theta_{t}\left(*\right)>y_{t}\left(i\right)|F_{t-1}\right)$. For any t∈T(τ) and $i\neq i_{t}^{*}$,*

$$\sum_{t\in T(\gamma )}E\left[\frac{1-p_{i,t}}{p_{i,t}}1\left\{i_{t}=i_{t}^{*},\theta_{t}\left(i\right)<y_{t}\left(i\right)\right\}\right]\leq \left(e^{11}+9+3\log\tau \right)\frac{T}{\tau }L_{2}\left(\tau \right).$$

This key lemma helps bound the regret that comes from the under-estimation of the optimal arm (Step 5 in the proof of DS-TS) which is similar to Lemma 3. It can be proved by Lemma A2, which transforms the probability of selecting the *i*th arm into the probability of selecting the optimal arm $i_{t}^{*}$. The detailed proofs can be found in Appendix B.6.

The rest of the proof is nearly identical to the proof of Theorem 1.

## 6. Experiments

In this section, we empirically compare the performance of our method with state-of-the-art algorithms on Bernoulli and Gaussian reward distributions (our code is available at https://github.com/qh1874/TS_NonStationary (accessed on 14 August 2024)). Specifically, we compare DS-TS and SW-TS with Thompson sampling to evaluate the improvement obtained thanks to the employment of the discounted factor γ and sliding window τ. We also compare our method with the UCB method, DS-UCB and SW-UCB [12] to evaluate the effect of Thompson sampling and UCB. Furthermore, we compare our method with some novel and efficient algorithms such as CUSUM [7], M-UCB [8] and SW-LB-SDA [15]. Note that SW-LB-SDA is not applicable to Gaussian reward distributions with unknown variance. We measure the performance of each algorithm with the cumulative expected regret defined in Equation (1). The expected regret is averaged over 100 independently runs. The 95% confidence interval is obtained by performing 100 independent runs and is depicted as a semi-transparent region in the figure.

### 6.1. Gaussian Arms

#### 6.1.1. Experimental Setting for Gaussian Arms

**Figure 2:**
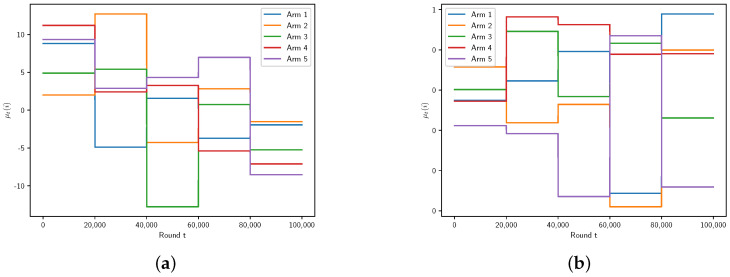
$K=5,B_{T}=5$. Gaussian arms (**a**), Bernoulli arms (**b**).

We fix the time horizon as *T* = 100,000. The mean and standard deviation are drawn from distributions $N(0,5^{2})$ and U(1,5). For Gaussian rewards, we conduct two experiments. In the first experiment, we split the time horizon into five phases and use a number of arms K=5. While in the second experiment, we split the time horizon into 10 phases and use a number of arms K=10. Figure 2 depicts the expected rewards for Gaussian arms and Bernoulli arms with K=5 and $B_{T}=5$.

The analysis of SW-UCB and DS-UCB is conducted under the bounded reward assumption, but the algorithms can adapt to Gaussian scenarios. To achieve reasonable performance, it is necessary to adjust the discounted factor and the sliding-window appropriately. We use the settings recommended in [15], where $\tau =2\left(1+2\sigma \right)\sqrt{T\log\left(T\right)/B_{T}}$ for SW-UCB and $\gamma =1-1/\left(4\left(1+2\sigma \right)\right)\sqrt{B_{T}/T}$ for DS-UCB.

#### 6.1.2. Results

**Figure 3:**
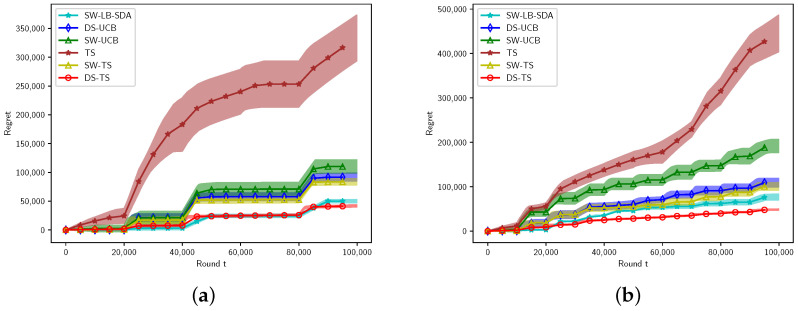
Gaussian arms. (**a**) $K=5,B_{T}=5$. (**b**) $K=10,B_{T}=10$.

Figure 3 illustrates the performance of these algorithms for Gaussian rewards under two different settings. Notably, CUSUM and M-UCB are not applicable to Gaussian rewards: CUSUM is designed for Bernoulli distributions, while M-UCB assumes bounded distributions. The discounted methods tend to perform better than sliding-window methods in Gaussian rewards.

Among these algorithms, only our algorithms and SW-LB-SDA provide regret analysis for unbounded rewards. Our algorithm (DS-TS) and SW-LB-SDA have demonstrated highly competitive experimental performance.

### 6.2. Bernoulli Arms

#### 6.2.1. Experimental Setting for Bernoulli Arms

The time horizon is set as *T* = 100,000. We split the time horizon into 5,10 phases of equal length and use a number of arms K={5,10}, respectively.

For Bernoulli rewards, the expected value $\mu_{t}\left(i\right)$ of each arm *i* is drawn from a uniform distribution over [0,1]. In the stationary phase, the rewards distributions remain unchanged. The Bernoulli arms for each phase are generated as $\mu_{t}\left(i\right)∼U\left(0,1\right).$

For a Bernoulli distribution, we modify the Thompson sampling (step 3) in our algorithm as $\theta_{t}\left(i\right)∼N\left({\hat{\mu }}_{t}\left(\gamma ,i\right),\frac{1}{N_{t}\left(\gamma ,i\right)}\right)$ and $\theta_{t}\left(i\right)∼N\left({\hat{\mu }}_{t}\left(\tau ,i\right),\frac{1}{N_{t}\left(\tau ,i\right)}\right)$. Based on Corollaries 1 and 2, we set $\gamma =1-\sqrt{\frac{B_{T}}{T\log T}}$ and $\tau =\sqrt{T/B_{T}}\log T$. To allow for fair comparison, DS-UCB uses the discount factor $\gamma =1-\sqrt{B_{T}/T}/4$, SW-UCB uses the sliding window $\tau =2\sqrt{T\log T/B_{T}}$ suggested by [12]. Based on [15], we set $\tau =2\sqrt{T\log\left(T\right)/B_{T}}$ for LB-SDA. For changepoint detection algorithm M-UCB, we set $w=800,b=\sqrt{w/2\log(2KT^{2})}$ as suggested by [8]. But we set the amount of exploration as $\gamma =\sqrt{KB_{T}\log\left(T\right)/T}$. In practice, it has been found that using this value instead of the one guaranteed in [8] will improve empirical performance [15]. For CUSUM, following from [7], we set $\alpha =\sqrt{B_{T}/T\log\left(T/B_{T}\right)}$ and $h=\log(T/B_{T})$. For our experiment settings, we choose M=50,ϵ=0.05.

#### 6.2.2. Results

**Figure 4:**
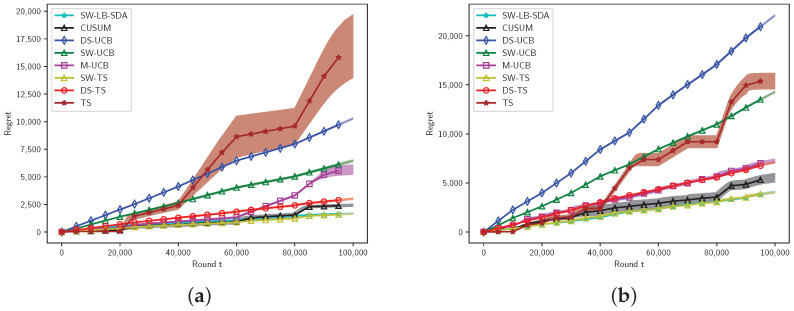
Bernoulli arms. Settings with $K=5,B_{T}=5$ (**a**), $K=10,B_{T}=10$ (**b**).

Figure 4 presents the results for Bernoulli arms in abruptly changing settings. It can be observed that our method (SW-TS) and SW-LB-SDA exhibit almost identical performance. Thompson sampling, designed for stationary MAB problems, shows significant oscillations at the breakpoints. The changepoint detection algorithm CUSUM [7] also shows competitive performance. Note that our experiment does not satisfy the detectability assumption of CUSUM. As the number of arms and breakpoints increase, the performance of UCB-class algorithms (DS-UCB, SW-UCB) declines, while two TS-based algorithms (DS-TS, SW-TS) still work well.

#### 6.2.3. Storage and Compute Cost

These algorithms can be divided into three class: UCB, TS and SW-LB-SDA. At each round, UCB-class and TS-class algorithms require O(K) storage and spend O(K) time complexity for computational cost. However, for round *T*, SW-LB-SDA require $O(K{\left(\log T\right)}^{2})$ storage and spend O(KlogT) time cost. Although the experimental performance of SW-LB-SDA is similar to our algorithms, our algorithm has less storage space and lower computational complexity.

### 6.3. Different Variance

The non-stationary setting has greater noise for estimation as compared to the stationary setting. Intuitively, TS with standard variance for the non-stationary setting should have worse regret as compared to the one with a larger variance. In this subsection, we conduct some experiments to verify this point. Table 1 shows the experimental results. For TS and the SW-TS algorithm, larger variance does indeed lead to smaller regret. This conclusion does not hold for DS-TS. We believe this does not contradict the above conclusion, because for DS-TS, the discount factor plays a more important role. If an arm has not been selected for some rounds, then $N_{t}\left(\gamma ,i\right)$ will be small ($N_{t}\left(\gamma ,i\right)$ can be close to 0, while if $N_{t}\left(\tau ,i\right)$ for SW-TS is greater than 1 or equal to 0), then $\frac{\sigma }{N_{t}\left(\gamma ,i\right)}$ has already become large, ensuring exploration performance. Therefore, DS-TS achieves the minimum regret. However, $\frac{2\sigma }{N_{t}\left(\gamma ,i\right)}$ may be too large, leading to excessive exploration and thus reducing regret compared to $\frac{\sigma }{N_{t}\left(\gamma ,i\right)}$.

## 7. Conclusions

In this paper, we analyze the regret upper bound of the TS algorithm with an uninformative prior in non-stationary settings, filling a research gap in this field. Our approach builds upon previous works while tackling two key challenges specific to non-stationary environments: under-estimation of the optimal arm and the inability of DS-TS algorithm to fully forget previous information. Finally, we conduct some experiments to verify the theory results. Below we discuss the results and propose directions for future research.

(1) The standard posterior update rule for Thompson sampling has a sampling variance as $\frac{\sigma^{2}}{N}$. We use $\frac{4\sigma^{2}}{N}$ only for ease of analysis. While this discrepancy is significant only for relatively small values of *N*, it would be valuable to develop proof techniques that leverage the variance of standard Bayesian updates.

(2) Our regret upper bound includes an additional logarithmic term compared to DS-UCB and SW-UCB, along with coefficients of $e^{17}$ and $e^{11}$. It would be interesting to explore whether the additional logarithm and large coefficients are intrinsic to DS-TS and SW-TS algorithms or are a limitation of our analysis.

## Figures and Tables

**Table 1:** Settings with *T* = 100,000, $B_{T}$ = 5, *K* = 5 for Gaussian arms. The mean and standard deviation are drawn from distributions $N(0,5^{2})$ and U(1,5). We set σ=5.

Algorithms	TS		DS-TS		SW-TS	
**std**	$\frac{\sigma }{N}$	$\frac{2\sigma }{N}$	$\frac{\sigma }{N}$	$\frac{2\sigma }{N}$	$\frac{\sigma }{N}$	$\frac{2\sigma }{N}$
Regret	333,835	305,064	41,790	52,909	83,731	83,150

## Data Availability

Our code is available at https://github.com/qh1874/TS_NonStationary (accessed on 14 August 2024).

## Appendix A. Facts and Lemmas

Garivier and Moulines [12] has derived a Hoeffding-type inequality for self-normalized means with a random number of summands. Their bound is for bounded distribution. Leveraging the properties of σ-subGaussian distributions, we have the following bound for σ-subGaussian. Recall that

$$N_{t}\left(\gamma ,i\right)=\sum_{j=1}^{t}\gamma^{t-j}1\left\{i_{j}=i\right\},{\hat{\mu }}_{t}\left(\gamma ,i\right)=\frac{1}{N_{t}\left(\gamma ,i\right)}\sum_{j=1}^{t}\gamma^{t-j}X_{j}\left(i\right)1\left\{i_{j}=i\right\}$$

$${\ddot{\mu }}_{t}\left(\gamma ,i\right)=\frac{1}{N_{t}\left(\gamma ,i\right)}\sum_{j=1}^{t}\gamma^{t-j}1\left\{i_{j}=i\right\}\mu_{j}\left(i\right)$$

**Lemma** **A1.**
*Let t∈T(γ),δ>0,*

$$P\left(\frac{N_{t}\left(\gamma ,i\right)\left(\hat{\mu }\left(\gamma ,i\right)-\ddot{\mu }\left(\gamma ,i\right)\right)}{\sqrt{N_{t}\left(\gamma^{2},i\right)}}>\delta \right)\leq \log\left(\frac{1}{1-\gamma }\right)\exp\left(-\frac{3\delta^{2}}{8\sigma^{2}}\right).$$

*Let t∈T(τ),δ>0,*

$$P\left(\sqrt{N_{t}\left(\tau ,i\right)}\left(\hat{\mu }\left(\tau ,i\right)-\mu_{t}\left(i\right)\right)>\delta \right)\leq \log\tau \exp\left(-\frac{3\delta^{2}}{8\sigma^{2}}\right),$$

The following inequality is the anti-concentration and concentration bound for Gaussian distributed random variables.

**Fact** **1** ([35])**.** *For a Gaussian distributed random variable X with mean μ and variance $\sigma^{2}$, for any a>0*

(A1) $$\frac{1}{\sqrt{2\pi }}\frac{a}{1+a^{2}}e^{-a^{2}/2}\leq P\left(X-\mu >a\sigma \right)\leq \frac{1}{a+\sqrt{a^{2}+4}}e^{-a^{2}/2}$$

*Since $\frac{1}{a+\sqrt{a^{2}+4}}\leq \frac{1}{2}$, we also have the following well-known result:*

$$P\left(X-\mu >a\sigma \right)\leq \frac{1}{2}e^{-a^{2}/2}$$

The following lemma is adapted from [32] and is often used in the analysis of Thompson sampling, which can transform the probability of selecting the *i*th arm into the probability of selecting the optimal arm $i_{t}^{*}$.

**Lemma** **A2.**
*Let $p_{i,t}=P\left(\theta_{t}\left(*\right)>y_{t}\left(i\right)|F_{t-1}\right)$. For any A>0, $i\neq i_{t}^{*}$,*

$$P\left(i_{t}=i,\theta_{t}\left(i\right)<y_{t}\left(i\right)|F_{t-1}\right)\leq \frac{\left(1-p_{i,t}\right)}{p_{i,t}}P\left(i_{t}=i_{t}^{*},\theta_{t}\left(i\right)<y_{t}\left(i\right)|F_{t-1}\right)$$

**Lemma** **A3** ([12])**.** *For any i∈{1,…,K}, γ∈(0,1) and A>0,*

$$\sum_{t=1}^{T}1\left\{i_{t}=i,N_{t}\left(\gamma ,i\right)<A\right\}\leq \left\lceil T\left(1-\gamma \right)\right\rceil A\gamma^{-1/(1-\gamma )},$$

$$\sum_{t=1}^{T}1\left\{i_{t}=i,N_{t}\left(\tau ,i\right)<A\right\}\leq \left\lceil \frac{T}{\tau }\right\rceil A.$$

## Appendix B. Detailed Proofs of Lemmas and Theorems

### Appendix B.1. Proof of Lemma 1

Recall that ${\ddot{\mu }}_{t}\left(\gamma ,i\right)=\frac{1}{N_{t}\left(\gamma ,i\right)}\sum_{j=1}^{t}\gamma^{t-j}1\left\{i_{j}=i\right\}\mu_{j}\left(i\right)$. Since ${\ddot{\mu }}_{t}\left(\gamma ,i\right)$ is a convex combination of elements $\mu_{j}\left(i\right),j=1,\ldots ,t$, we have

(A2) $$|\mu_{t}\left(i\right)-{\ddot{\mu }}_{t}\left(\gamma ,i\right)|\leq \Delta_{max}^{T}$$

We can write $\mu_{t}\left(i\right)$ as $\mu_{t}\left(i\right)=\frac{1}{N_{t}\left(\gamma ,i\right)}\sum_{j=1}^{t}\gamma^{t-j}1\left\{i_{j}=i\right\}\mu_{t}\left(i\right)$. Thus, we have

$$|\mu_{t}\left(i\right)-{\ddot{\mu }}_{t}\left(\gamma ,i\right)|=\frac{1}{N_{t}\left(\gamma ,i\right)}|\sum_{j=1}^{t}\gamma^{t-j}\left(\mu_{j}\left(i\right)-\mu_{t}\left(i\right)\right)1\left\{i_{j}=i\right\}|.$$

Recall that $T\left(\gamma \right)=\{t\leq T:\forall s\in \left(t-D\left(\gamma \right),t\right],\mu_{s}\left(\cdot \right)=\mu_{t}\left(\cdot \right)\}$. If t∈T(γ), we have $\mu_{j}\left(i\right)=\mu_{t}\left(i\right),\forall j\in \left(t-D\left(\gamma \right),t\right).$

Therefore, ∀t∈T(γ), we have

$$\begin{array}{cc} |\mu_{t}\left(i\right)-{\ddot{\mu }}_{t}\left(\gamma ,i\right)| & =\frac{1}{N_{t}\left(\gamma ,i\right)}|\sum_{j=1}^{t-D(\gamma )}\gamma^{t-j}\left(\mu_{j}\left(i\right)-\mu_{t}\left(i\right)\right)1\left\{i_{j}=i\right\}|\\ & \leq \frac{\Delta_{max}^{T}}{N_{t}\left(\gamma ,i\right)}\sum_{j=1}^{t-D(\gamma )}\gamma^{t-j}1\left\{i_{j}=i\right\}\\ & =\frac{\Delta_{max}^{T}}{N_{t}\left(\gamma ,i\right)}\gamma^{D(\gamma )}N_{t-D(\gamma )}\left(\gamma ,i\right)\\ & \leq \frac{\Delta_{max}^{T}\gamma^{D(\gamma )}}{N_{t}\left(\gamma ,i\right)\left(1-\gamma \right)} \end{array}$$

where the last inequality follows from $N_{t-D(\gamma )}\left(\gamma ,i\right)\leq \frac{1}{1-\gamma }$.

If $\frac{\gamma^{D(\gamma )}}{N_{t}\left(\gamma ,i\right)\left(1-\gamma \right)}<1$, $\frac{\gamma^{D(\gamma )}}{N_{t}\left(\gamma ,i\right)\left(1-\gamma \right)}<\sqrt{\frac{\gamma^{D(\gamma )}}{N_{t}\left(\gamma ,i\right)\left(1-\gamma \right)}}$, we have

$$|\mu_{t}\left(i\right)-{\ddot{\mu }}_{t}\left(\gamma ,i\right)|\leq \Delta_{max}^{T}\sqrt{\frac{\gamma^{D(\gamma )}}{N_{t}\left(\gamma ,i\right)\left(1-\gamma \right)}}.$$

If $\frac{\gamma^{D(\gamma )}}{N_{t}\left(\gamma ,i\right)\left(1-\gamma \right)}\geq 1$, from Equation (A2), we also have

$$|\mu_{t}\left(i\right)-{\ddot{\mu }}_{t}\left(\gamma ,i\right)|\leq \Delta_{max}^{T}\leq \Delta_{max}^{T}\sqrt{\frac{\gamma^{D(\gamma )}}{N_{t}\left(\gamma ,i\right)\left(1-\gamma \right)}}.$$

By the definition of $D\left(\gamma \right)=\frac{\log({\left(\frac{\sigma }{\Delta_{max}^{T}}\right)}^{2}{\left(1-\gamma \right)}^{2}\log\frac{1}{1-\gamma })}{\log\gamma }$,

$$|\mu_{t}\left(i\right)-{\ddot{\mu }}_{t}\left(\gamma ,i\right)|\leq \sigma \sqrt{\frac{\left(1-\gamma \right)\log\frac{1}{1-\gamma }}{N_{t}\left(\gamma ,i\right)}}$$

### Appendix B.2. Proof of Lemma 2

From the definition of $n,A\left(\gamma \right),U_{t}\left(\gamma ,i\right)$ in Equation (4), we can obtain

(A3) $$U_{t}\left(\gamma ,i\right)=\frac{\sqrt{1-\gamma }\Delta_{T}\left(i\right)}{n}\sqrt{\frac{A(\gamma )}{N_{t}\left(\gamma ,i\right)}}.$$

If $N_{t}\left(\gamma ,i\right)>A\left(\gamma \right)$, $U_{t}\left(\gamma ,i\right)<\frac{\sqrt{1-\gamma }}{n}\Delta_{T}\left(i\right)$. Thus, we have

(A4) $$\frac{\Delta_{t}\left(i\right)}{3}-U_{t}\left(\gamma ,i\right)>\frac{\Delta_{T}\left(i\right)}{3}-\frac{\sqrt{1-\gamma }}{n}\Delta_{T}\left(i\right)=\frac{2\sqrt{2}}{n}\Delta_{T}\left(i\right).$$

Therefore,

(A5) $$\begin{array}{cc} \; & P({\hat{\mu }}_{t}\left(\gamma ,i\right)>\mu_{t}\left(i\right)+\frac{\Delta_{t}\left(i\right)}{3},N_{t}\left(\gamma ,i\right)>A\left(\gamma \right))\\ \; & \;\overset{\left(a\right)}{\leq }P\left({\hat{\mu }}_{t}\left(\gamma ,i\right)-{\ddot{\mu }}_{t}\left(\gamma ,i\right)>\frac{\Delta_{t}\left(i\right)}{3}-U_{t}\left(\gamma ,i\right),N_{t}\left(\gamma ,i\right)>A\left(\gamma \right)\right)\\ \; & \;\overset{\left(b\right)}{\leq }P\left({\hat{\mu }}_{t}\left(\gamma ,i\right)-{\ddot{\mu }}_{t}\left(\gamma ,i\right)>\frac{2\sqrt{2}}{n}\Delta_{T}\left(i\right),N_{t}\left(\gamma ,i\right)>A\left(\gamma \right)\right)\\ \; & \;\overset{\left(c\right)}{\leq }P\left(\frac{N_{t}\left(\gamma ,i\right)\left({\hat{\mu }}_{t}\left(\gamma ,i\right)-{\ddot{\mu }}_{t}\left(\gamma ,i\right)\right)}{\sqrt{N_{t}\left(\gamma^{2},i\right)}}>\frac{2\sqrt{2}}{n}\Delta_{T}\left(i\right)\sqrt{A(\gamma )}\right)\\ \; & \;\overset{\left(d\right)}{\leq }\log\frac{1}{1-\gamma }\exp\left(-\frac{3{\left(\Delta_{T}\left(i\right)\right)}^{2}}{n^{2}\sigma^{2}}A\left(\gamma \right)\right)\\ \; & \;\leq {\left(1-\gamma \right)}^{3}\log\frac{1}{1-\gamma } \end{array}$$

where (a) uses Lemma 1, (b) uses Equation (A4), (c) follows from $N_{t}\left(\gamma ,i\right)>N_{t}\left(\gamma^{2},i\right)$, (d) uses Lemma A1.

Since $\left(1-\gamma \right)\log\frac{1}{1-\gamma }\leq \frac{1}{e}<1$, this ends the proof.

### Appendix B.3. Proof of Lemma 3

This proof is adapted from [32] for the stationary settings. However, there are some technical problems that are difficult to overcome in non-stationary settings. The tricky problem is to lower bound the probability of the mean’s estimation of optimal arm Equation (A9). By designing the function $U_{t}\left(\gamma ,i\right)$ and decomposing the regret to use Lemma A3 again, we solve this challenge. We use blue font to emphasize the techniques used in the proof.

The proof is in three steps.

**Step 1:** We first prove that $E[\frac{1}{p_{i,t}}]$ has an upper bound independent of *t*.

Define a Bernoulli experiment as sampling from $N({\hat{\mu }}_{t}\left(*\right),\frac{4\sigma^{2}}{N_{t}\left(\gamma ,*\right)})$, where success implies that $\theta_{t}\left(*\right)>y_{t}\left(i\right)$. Let $G_{t}$ denote the number of experiments performed when the event $\left\{\theta_{t}\left(*\right)>y_{t}\left(i\right)\right\}$ first occurs. Then,

$$E\left[\frac{1}{p_{i,t}}\right]=E\left[E\left[G_{t}|F_{t-1}\right]\right]=E\left[G_{t}\right]$$

Let $z=\sqrt{\log r}+\frac{1}{2}$ (r≥1 is an integer ) and let ${\mathrm{MAX}}_{r}$ denote the maximum of *r* independent Bernoulli experiments. Then,

(A6) $$\begin{array}{cc} P(G_{t}\leq r) & \geq P({\mathrm{MAX}}_{r}>{\hat{\mu }}_{t}\left(*\right)+\frac{z\cdot 2\sigma }{\sqrt{N_{t}\left(\gamma ,*\right)}}\geq y_{t}\left(i\right))\\ \; & =E\left[E\left[1\left\{{\mathrm{MAX}}_{r}>{\hat{\mu }}_{t}\left(*\right)+\frac{z\cdot 2\sigma }{\sqrt{N_{t}\left(\gamma ,*\right)}}\geq y_{t}\left(i\right)\right\}|F_{t-1}\right]\right]\\ \; & =E\left[1\left\{{\hat{\mu }}_{t}\left(*\right)+\frac{z\cdot 2\sigma }{\sqrt{N_{t}\left(\gamma ,*\right)}}\geq y_{t}\left(i\right)\right\}\cdot P\left({\mathrm{MAX}}_{r}>{\hat{\mu }}_{t}\left(*\right)+\frac{z\cdot 2\sigma }{\sqrt{N_{t}\left(\gamma ,*\right)}}|F_{t-1}\right)\right] \end{array}$$

Using Fact A1,

(A7) $$\begin{array}{cc} P({\mathrm{MAX}}_{r}>{\hat{\mu }}_{t}\left(*\right)+\frac{z\cdot 2\sigma }{\sqrt{N_{t}\left(\gamma ,*\right)}}|F_{t-1})\; & \geq 1-{\left(1-\frac{1}{\sqrt{2\pi }}\frac{z}{z^{2}+1}e^{-z^{2}/2}\right)}^{r}\\ \; & =1-{\left(1-\frac{1}{\sqrt{2\pi }}\frac{\sqrt{\log r}+\frac{1}{2}}{{\left(\sqrt{\log r}+\frac{1}{2}\right)}^{2}+1}\frac{e^{-1/4-\sqrt{\log r}/2}}{\sqrt{r}}\right)}^{r}\\ \; & \geq 1-e^{-\frac{\sqrt{r}e^{-\sqrt{\log r}/2}}{e^{0.25}\sqrt{2\pi }\left(\sqrt{\log r}+1\right)}} \end{array}$$

For any $r\geq e^{17}$, $e^{-\frac{\sqrt{r}e^{-\sqrt{\log r}/2}}{e^{0.25}\sqrt{2\pi }\left(\sqrt{\log r}+1\right)}}\leq \frac{1}{r^{2}}$. Hence, for any $r\geq e^{17}$,

$$P\left({\mathrm{MAX}}_{r}>{\hat{\mu }}_{t}\left(*\right)+\frac{z\cdot 2\sigma }{\sqrt{N_{t}\left(\gamma ,*\right)}}|F_{t-1}\right)\geq 1-\frac{1}{r^{2}}.$$

Therefore, for any $r\geq e^{17}$,

$$P\left(G_{t}\leq r\right)\geq \left(1-\frac{1}{r^{2}}\right)P\left({\hat{\mu }}_{t}\left(*\right)+\frac{z}{\sqrt{N_{t}\left(\gamma ,*\right)}}\geq y_{t}\left(i\right)\right)$$

Next, we apply Lemma A1 to lower bound $P({\hat{\mu }}_{t}\left(*\right)+\frac{z\cdot 2\sigma }{\sqrt{N_{t}\left(\gamma ,*\right)}}\geq y_{t}\left(i\right))$.

(A8) $$\begin{array}{cc} P({\hat{\mu }}_{t}\left(*\right)+\frac{z\cdot 2\sigma }{\sqrt{N_{t}\left(\gamma ,*\right)}}\geq y_{t}\left(i\right)) & \geq 1-P({\hat{\mu }}_{t}\left(*\right)+\frac{z\cdot 2\sigma }{\sqrt{N_{t}\left(\gamma ,*\right)}}\leq \mu_{t}\left(*\right))\\ \; & \geq 1-P({\hat{\mu }}_{t}\left(*\right)-{\ddot{\mu }}_{t}\left(*\right)\leq U_{t}\left(\gamma ,*\right)-\frac{z\cdot 2\sigma }{\sqrt{N_{t}\left(\gamma ,*\right)}}) \end{array}$$

Since $U_{t}\left(\gamma ,*\right)=\frac{\sigma \sqrt{\left(1-\gamma \right)\log\frac{1}{1-\gamma }}}{\sqrt{N_{t}\left(\gamma ,*\right)}},z=\log r+\frac{1}{2}$,

$$U_{t}\left(\gamma ,*\right)-\frac{z\cdot 2\sigma }{\sqrt{N_{t}\left(\gamma ,*\right)}}=\frac{\sigma \sqrt{\left(1-\gamma \right)\log\frac{1}{1-\gamma }}-\sigma -2\sigma \sqrt{\log r}}{\sqrt{N_{t}\left(\gamma ,*\right)}}<-\frac{2\sigma \sqrt{\log r}}{\sqrt{N_{t}\left(\gamma ,*\right)}}.$$

Then, we have

(A9) $$\begin{array}{cc} P({\hat{\mu }}_{t}\left(*\right)+\frac{z\cdot 2\sigma }{\sqrt{N_{t}\left(\gamma ,*\right)}}\geq y_{t}\left(i\right))\; & \geq 1-P({\hat{\mu }}_{t}\left(*\right)-{\ddot{\mu }}_{t}\left(*\right)<-\frac{2\sigma \sqrt{\log r}}{\sqrt{N_{t}\left(\gamma ,*\right)}})\\ \; & \geq 1-\log\left(\frac{1}{1-\gamma }\right)e^{-\frac{3}{2}\log r}\\ \; & \geq 1-\log\frac{1}{1-\gamma }\frac{1}{r^{1.5}}. \end{array}$$

Substituting, for any $r>e^{17}$,

(A10) $$P\left(G_{t}\leq r\right)\geq 1-\log\frac{1}{1-\gamma }\frac{1}{r^{1.5}}-\frac{1}{r^{2}}$$

Therefore,

(A11) $$\begin{array}{cc} E[G_{t}] & =\sum_{r=0}^{\infty }P\left(G_{t}\geq r\right)\\ \; & \leq 1+e^{17}+\sum_{r>e^{17}}\left(\log\frac{1}{1-\gamma }\frac{1}{r^{1.5}}+\frac{1}{r^{2}}\right)\\ \; & \leq e^{17}+3+3\log\frac{1}{1-\gamma } \end{array}$$

This proves a bound of $E\left[\frac{1}{p_{i,t}}\right]\leq e^{17}+3+3\log\frac{1}{1-\gamma }$ independent of *t*.

**Step 2:** Define $L\left(\gamma \right)=\frac{1152\log\left(\frac{1}{1-\gamma }+e^{17}\right)\sigma^{2}}{{\left(\Delta_{T}\left(i\right)\right)}^{2}}$. We consider the upper bound of $E[\frac{1}{p_{i,t}}]$ when $N_{t}\left(\gamma ,*\right)>L\left(\gamma \right)$.

(A12) $$\begin{array}{cc} P(G_{t}\leq r) & \geq P({\mathrm{MAX}}_{r}>{\hat{\mu }}_{t}\left(*\right)+\frac{z\cdot 2\sigma }{\sqrt{N_{t}\left(\gamma ,*\right)}}-\frac{\Delta_{t}\left(i\right)}{6}\geq y_{t}\left(i\right))\\ \; & =E[1\{{\hat{\mu }}_{t}\left(*\right)+\frac{z\cdot 2\sigma }{\sqrt{N_{t}\left(\gamma ,*\right)}}-\frac{\Delta_{t}\left(i\right)}{6}\geq y_{t}\left(i\right)\}\\ \; & \;\cdot P({\mathrm{MAX}}_{r}>{\hat{\mu }}_{t}\left(*\right)+\frac{z\cdot 2\sigma }{\sqrt{N_{t}\left(\gamma ,*\right)}}-\frac{\Delta_{t}\left(i\right)}{6}|F_{t-1})] \end{array}$$

Now, since $N_{t}\left(\gamma ,*\right)>L\left(\gamma \right)$,$\frac{1}{\sqrt{N_{t}\left(\gamma ,*\right)}}<\frac{\Delta_{t}\left(i\right)}{48\sqrt{\log(\frac{1}{1-\gamma }+e^{17})}\sigma }$. Therefore, for any $r\leq {\left(\frac{1}{1-\gamma }+e^{17}\right)}^{2}$,

$$\frac{z\cdot 2\sigma }{\sqrt{N_{t}\left(\gamma ,*\right)}}-\frac{\Delta_{t}\left(i\right)}{6}=\frac{2\sigma \sqrt{\log r}+\sigma }{\sqrt{N_{t}\left(\gamma ,*\right)}}-\frac{\Delta_{t}\left(i\right)}{6}\leq -\frac{\Delta_{t}\left(i\right)}{12}.$$

Using Fact A1,

$$P\left(\theta_{t}\left(i\right)>{\hat{\mu }}_{t}\left(i\right)-\frac{\Delta_{t}\left(i\right)}{12}|F_{t-1}\right)\leq 1-\frac{1}{2}e^{-\frac{N_{t}\left(\gamma ,*\right)}{4\sigma^{2}}\frac{\Delta_{t}{\left(i\right)}^{2}}{288}}\geq 1-\frac{1}{2(1/\left(1-\gamma \right)+e^{17})}.$$

This implies

$$P\left({\mathrm{MAX}}_{r}>{\hat{\mu }}_{t}\left(*\right)+\frac{z}{\sqrt{N_{t}\left(\gamma ,*\right)}}-\frac{\Delta_{t}\left(i\right)}{6}|F_{t-1}\right)\geq 1-\frac{1}{2^{r}{\left(1/\left(1-\gamma \right)+e^{17}\right)}^{r}}.$$

Also, apply the self-normalized Hoeffding-type inequality,

$$\begin{array}{cc} P({\hat{\mu }}_{t}\left(*\right)+\frac{z\cdot 2\sigma }{\sqrt{N_{t}\left(\gamma ,*\right)}}-\frac{\Delta_{t}\left(i\right)}{6}\geq y_{t}\left(i\right)) & \geq 1-P({\hat{\mu }}_{t}\left(*\right)\leq \mu_{t}\left(*\right)-\frac{\Delta_{t}\left(i\right)}{6})\\ & \geq 1-P({\hat{\mu }}_{t}\left(*\right)-{\ddot{\mu }}_{t}\left(*\right)\geq -U_{t}\left(\gamma ,*\right)+\frac{\Delta_{t}\left(i\right)}{6})\\ & >1-P({\hat{\mu }}_{t}\left(*\right)-{\ddot{\mu }}_{t}\left(*\right)\geq \frac{\Delta_{T}\left(i\right)}{8}\sqrt{\frac{L(\gamma )}{N_{t}\left(\gamma ,*\right)}})\\ & \geq 1-\log\left(\frac{1}{1-\gamma }+e^{17}\right)\frac{1}{{\left(1/\left(1-\gamma \right)+e^{17}\right)}^{3}}. \end{array}$$

Let $\gamma^{\prime }={\left(\frac{1}{1-\gamma }+e^{17}\right)}^{2}$. Therefore, for any $1\leq r\leq \gamma^{\prime }$,

$$P\left(G_{t}\leq r\right)\geq 1-\frac{1}{2^{r}{\gamma^{\prime }}^{r/2}}-\log\left(\frac{1}{1-\gamma }+e^{17}\right)\frac{1}{{\gamma^{\prime }}^{1.5}}.$$

When $r\geq \gamma^{\prime }>e^{17}$, we can use Equation (A10) to obtain

$$P\left(G_{t}\leq r\right)\geq 1-\log\frac{1}{1-\gamma }\frac{1}{r^{1.5}}-\frac{1}{r^{2}}$$

Combining these results

$$\begin{array}{cc} E[G_{t}] & \leq \sum_{r=0}^{\infty }P\left(G_{t}\geq r\right)\\ & \leq 1+\sum_{r=1}^{\gamma^{\prime }}P\left(G_{t}\geq r\right)+\sum_{r=\gamma^{\prime }}^{\infty }P\left(G_{t}\geq r\right)\\ & \leq 1+\sum_{r=1}^{\gamma^{\prime }}\left(\frac{1}{2^{r}{\gamma^{\prime }}^{r/2}}+\log\left(\frac{1}{1-\gamma }+e^{17}\right)\frac{1}{{\gamma^{\prime }}^{1.5}}\right)+\sum_{r=\gamma^{\prime }}^{\infty }\left(\log\frac{1}{1-\gamma }\frac{1}{r^{1.5}}+\frac{1}{r^{2}}\right)\\ & \leq 1+\frac{1}{2\sqrt{\gamma^{\prime }}}+\log\left(\frac{1}{1-\gamma }+e^{17}\right)\frac{1}{\sqrt{\gamma^{\prime }}}+\frac{2}{\gamma^{\prime }}+\log\left(\frac{1}{1-\gamma }\right)\frac{3}{\sqrt{\gamma^{\prime }}}\\ & \leq 1+6\left(1-\gamma \right)\log\left(\frac{1}{1-\gamma }+e^{17}\right). \end{array}$$

Therefore, when $N_{t}\left(\gamma ,*\right)>L\left(\gamma \right)$, it holds that

$$E\left[\frac{1}{p_{i,t}}\right]-1=E\left[G_{t}\right]-1\leq 6\left(1-\gamma \right)\log\left(\frac{1}{1-\gamma }+e^{17}\right).$$

**Step 3:** Let $A\left(\gamma ,*\right)=\{t\in \left\{1,\ldots ,T\right\}:N_{t}\left(\gamma ,*\right)\leq L\left(\gamma \right)\}$.

(A13) $$\begin{array}{cc} \; & \sum_{t\in T(\gamma )}E\left[\frac{1-p_{i,t}}{p_{i,t}}1\left\{i_{t}=i_{t}^{*},\theta_{t}\left(i\right)<y_{t}\left(i\right)\right\}\right]\\ \; & \leq \left(\sum_{t\in T(\gamma )\cap A(\gamma ,*)}+\sum_{t\in T(\gamma )\setminus A(\gamma ,*)}\right)E\left[\frac{1-p_{i,t}}{p_{i,t}}1\left\{i_{t}=i_{t}^{*},\theta_{t}\left(i\right)<y_{t}\left(i\right)\right\}\right]\\ \; & \leq |\left\{t:i_{t}=i_{t}^{*},N_{t}\left(\gamma ,*\right)\leq L\left(\gamma \right)\right\}|\left(e^{17}+3+3\log\frac{1}{1-\gamma }\right)+\sum_{t\in T(\gamma )\setminus A(\gamma ,*)}E\left[\frac{1-p_{i,t}}{p_{i,t}}\right]\\ \; & \leq T\left(1-\gamma \right)L\left(\gamma \right)\gamma^{-1/(1-\gamma )}\left(e^{17}+3+3\log\frac{1}{1-\gamma }\right)+6T\left(1-\gamma \right)\log\left(\frac{1}{1-\gamma }+e^{17}\right)\\ \; & \leq \left(e^{17}+9+3\log\frac{1}{1-\gamma }\right)T\left(1-\gamma \right)L\left(\gamma \right)\gamma^{-1/(1-\gamma )}. \end{array}$$

### Appendix B.4. Larger Variance

In fact, Lemma A1 has a stricter upper bound as $\frac{\log\frac{1}{1-\gamma }}{\log(1+\eta )}\exp\left(-\frac{1}{2\sigma^{2}}\left(1-\frac{\eta^{2}}{16}\right)\right)$, where η∈(−1,4). Let η=2, then we obtain Lemma A1. Suppose the variance of Thompson sampling is $\frac{\xi \sigma^{2}}{N_{t}\left(\gamma ,i\right)}$. The lower bound of Equation (A9) becomes

$$\frac{\log\frac{1}{1-\gamma }}{\log(1+\eta )}\exp\left(-\frac{\xi \log r}{2}\left(1-\frac{\eta^{2}}{16}\right)\right).$$

To ensure that $E[G_{t}]$ in Equation (A11) has a finite upper bound, our analysis method requires the infinite series

$$\sum_{r=1}^{\infty }\exp\left(-\frac{\xi \log r}{2}\left(1-\frac{\eta^{2}}{16}\right)\right)=\sum_{r=1}^{\infty }\frac{1}{r^{\frac{\xi }{2}\left(1-\frac{\eta^{2}}{16}\right)}}$$

is convergent. Thus, we have

$$\xi >\frac{2}{1-\frac{\eta^{2}}{16}}>2,$$

i.e., the sampling variance needs to be strictly greater than $\frac{2\sigma^{2}}{N_{t}\left(\gamma ,i\right)}$.

### Appendix B.5. Proof of Lemma 4

Recall that $A\left(\tau \right)=\frac{72\log\left(\tau \right)\sigma^{2}}{{\left(\Delta_{T}\left(i\right)\right)}^{2}}$. Using Lemma A1, we have

(A14) $$\begin{array}{cc} P({\hat{\mu }}_{t}\left(\tau ,i\right)>x_{t}\left(i\right),N_{t}\left(\tau ,i\right)>A\left(\tau \right))\; & =P({\hat{\mu }}_{t}\left(\tau ,i\right)-\mu_{t}\left(i\right)>\frac{\Delta_{t}\left(i\right)}{3},N_{t}\left(\tau ,i\right)>A\left(\gamma \right))\\ \; & \leq P(\sqrt{N_{t}\left(\tau ,i\right)}\left({\hat{\mu }}_{t}\left(\tau ,i\right)-\mu_{t}\left(i\right)\right)>\frac{\Delta_{T}\left(i\right)}{3}\sqrt{A(\gamma )})\\ \; & \leq \log\tau \exp(-\frac{3{\left(\Delta_{T}\left(i\right)\right)}^{2}}{72\sigma^{2}}A\left(\gamma \right))\\ \; & \leq \frac{1}{\tau^{2}} \end{array}$$

### Appendix B.6. Proof of Lemma 5

The proof is similar to the proof of Lemma 3.

**Step 1:** We first prove that $E[\frac{1}{p_{i,t}}]$ has an upper bound independent of *t*.

Let $z=\sqrt{\log r}$ (r≥1 is an integer). Then,

(A15) $$\begin{array}{cc} P(G_{t}\leq r) & \geq P({\mathrm{MAX}}_{r}>{\hat{\mu }}_{t}\left(*\right)+\frac{z\cdot 2\sigma }{\sqrt{N_{t}\left(\tau ,*\right)}}\geq y_{t}\left(i\right))\\ \; & =E\left[1\left\{{\hat{\mu }}_{t}\left(*\right)+\frac{z\cdot 2\sigma }{\sqrt{N_{t}\left(\tau ,*\right)}}\geq y_{t}\left(i\right)\right\}P\left({\mathrm{MAX}}_{r}>{\hat{\mu }}_{t}\left(*\right)+\frac{z\cdot 2\sigma }{\sqrt{N_{t}\left(\tau ,*\right)}}|F_{t-1}\right)\right] \end{array}$$

Using Fact A1,

(A16) $$\begin{array}{cc} P({\mathrm{MAX}}_{r}>{\hat{\mu }}_{t}\left(*\right)+\frac{z\cdot 2\sigma }{\sqrt{N_{t}\left(\tau ,*\right)}}|F_{t-1})\; & \geq 1-{\left(1-\frac{1}{\sqrt{2\pi }}\frac{z}{z^{2}+1}e^{-z^{2}/2}\right)}^{r}\\ \; & =1-{\left(1-\frac{1}{\sqrt{2\pi }}\frac{\sqrt{\log r}}{{\left(\sqrt{\log r}\right)}^{2}+1}\frac{1}{\sqrt{r}}\right)}^{r}\\ \; & \geq 1-e^{-\frac{\sqrt{r}}{\sqrt{2\pi }\left(\sqrt{\log r}+1\right)}} \end{array}$$

For any $r\geq e^{11}$, $e^{-\frac{\sqrt{r}}{\sqrt{2\pi }\left(\sqrt{\log r}+1\right)}}\leq \frac{1}{r^{2}}$. Hence, for any $r\geq e^{11}$,

$$P\left({\mathrm{MAX}}_{r}>{\hat{\mu }}_{t}\left(*\right)+\frac{z\cdot 2\sigma }{\sqrt{N_{t}\left(\tau ,*\right)}}|F_{t-1}\right)\geq 1-\frac{1}{r^{2}}.$$

Therefore, for any $r\geq e^{11}$,

$$P\left(G_{t}\leq r\right)\geq \left(1-\frac{1}{r^{2}}\right)P\left({\hat{\mu }}_{t}\left(*\right)+\frac{z}{\sqrt{N_{t}\left(\tau ,*\right)}}\geq y_{t}\left(i\right)\right)$$

Next, we apply Lemma A1 to lower bound $P({\hat{\mu }}_{t}\left(*\right)+\frac{z\cdot 2\sigma }{\sqrt{N_{t}\left(\tau ,*\right)}}\geq y_{t}\left(i\right))$.

$$\begin{array}{cc} P({\hat{\mu }}_{t}\left(*\right)+\frac{z\cdot 2\sigma }{\sqrt{N_{t}\left(\tau ,*\right)}}\geq y_{t}\left(i\right)) & \geq 1-P({\hat{\mu }}_{t}\left(*\right)+\frac{z\cdot 2\sigma }{\sqrt{N_{t}\left(\tau ,*\right)}}\leq \mu_{t}\left(*\right))\\ & \geq 1-P({\hat{\mu }}_{t}\left(*\right)-\mu_{t}\left(*\right)<-\frac{2\sigma \sqrt{\log r}}{\sqrt{N_{t}\left(\tau ,*\right)}})\\ & \geq 1-\log\tau e^{-\frac{3}{2}\log r}\\ & =1-\log\tau \frac{1}{r^{1.5}}. \end{array}$$

Substituting, for any $r>e^{11}$,

(A17) $$P\left(G_{t}\leq r\right)\geq 1-\log\tau \frac{1}{r^{1.5}}-\frac{1}{r^{2}}$$

Therefore,

$$\begin{array}{cc} E[G_{t}] & =\sum_{r=0}^{\infty }P\left(G_{t}\geq r\right)\\ & \leq 1+e^{11}+\sum_{r>e^{11}}\left(\log\tau \frac{1}{r^{1.5}}+\frac{1}{r^{2}}\right)\\ & \leq e^{11}+3+3\log\tau \end{array}$$

This proves a bound of $E\left[\frac{1}{p_{i,t}}\right]\leq e^{11}+3+3\log\tau$ independent of *t*.

**Step 2:** Define $L\left(\tau \right)=\frac{1152\log\left(\tau +e^{11}\right)\sigma^{2}}{{\left(\Delta_{T}\left(i\right)\right)}^{2}}$. We consider the upper bound of $E[\frac{1}{p_{i,t}}]$ when $N_{t}\left(\tau ,*\right)>L\left(\tau \right)$.

(A18) $$\begin{array}{cc} P(G_{t}\leq r) & \geq P({\mathrm{MAX}}_{r}>{\hat{\mu }}_{t}\left(*\right)+\frac{z\cdot 2\sigma }{\sqrt{N_{t}\left(\tau ,*\right)}}-\frac{\Delta_{t}\left(i\right)}{6}\geq y_{t}\left(i\right))\\ \; & =E[1\{{\hat{\mu }}_{t}\left(*\right)+\frac{z\cdot 2\sigma }{\sqrt{N_{t}\left(\tau ,*\right)}}-\frac{\Delta_{t}\left(i\right)}{6}\geq y_{t}\left(i\right)\}\\ \; & \;\cdot P({\mathrm{MAX}}_{r}>{\hat{\mu }}_{t}\left(*\right)+\frac{z\cdot 2\sigma }{\sqrt{N_{t}\left(\tau ,*\right)}}-\frac{\Delta_{t}\left(i\right)}{6}|F_{t-1})] \end{array}$$

Now, since $N_{t}\left(\tau ,*\right)>L\left(\tau \right)$,$\frac{1}{\sqrt{N_{t}\left(\tau ,*\right)}}<\frac{\Delta_{t}\left(i\right)}{48\sqrt{\log(\tau +e^{11})}\sigma }$. Therefore, for any $r\leq {\left(\tau +e^{11}\right)}^{2}$,

$$\frac{z\cdot 2\sigma }{\sqrt{N_{t}\left(\tau ,*\right)}}-\frac{\Delta_{t}\left(i\right)}{6}=\frac{2\sigma \sqrt{\log r}+\sigma }{\sqrt{N_{t}\left(\tau ,*\right)}}-\frac{\Delta_{t}\left(i\right)}{6}\leq -\frac{\Delta_{t}\left(i\right)}{12}.$$

Using Fact A1,

$$P\left(\theta_{t}\left(i\right)>{\hat{\mu }}_{t}\left(i\right)-\frac{\Delta_{t}\left(i\right)}{12}|F_{t-1}\right)\leq 1-\frac{1}{2}e^{-\frac{N_{t}\left(\tau ,*\right)}{4\sigma^{2}}\frac{\Delta_{t}{\left(i\right)}^{2}}{288}}\geq 1-\frac{1}{2(\tau +e^{11})}.$$

This implies

$$P\left({\mathrm{MAX}}_{r}>{\hat{\mu }}_{t}\left(*\right)+\frac{z}{\sqrt{N_{t}\left(\tau ,*\right)}}-\frac{\Delta_{t}\left(i\right)}{6}|F_{t-1}\right)\geq 1-\frac{1}{2^{r}{\left(\tau +e^{11}\right)}^{r}}.$$

Also, apply Lemma A1,

$$\begin{array}{cc} P({\hat{\mu }}_{t}\left(*\right)+\frac{z}{\sqrt{N_{t}\left(\tau ,*\right)}}-\frac{\Delta_{t}\left(i\right)}{6}\geq y_{t}\left(i\right)) & \geq 1-P({\hat{\mu }}_{t}\left(*\right)-\mu_{t}\left(*\right)\geq \frac{\Delta_{t}\left(i\right)}{6})\\ & \geq 1-\log\left(\tau +e^{11}\right)\frac{1}{{\left(\tau +e^{11}\right)}^{3}}. \end{array}$$

Let $\tau^{\prime }={\left(\tau +e^{11}\right)}^{2}$. Therefore, for any $1\leq r\leq \tau^{\prime }$,

$$P\left(G_{t}\leq r\right)\geq 1-\frac{1}{2^{r}{\tau^{\prime }}^{r/2}}-\log\left(\tau +e^{11}\right)\frac{1}{{\tau^{\prime }}^{1.5}}.$$

When $r\geq \tau^{\prime }>e^{11}$, we can use Equation (A10) to obtain

$$P\left(G_{t}\leq r\right)\geq 1-\log\tau \frac{1}{r^{1.5}}-\frac{1}{r^{2}}$$

Combining these results,

$$\begin{array}{cc} E[G_{t}] & \leq \sum_{r=0}^{\infty }P\left(G_{t}\geq r\right)\\ & \leq 1+\sum_{r=1}^{\tau^{\prime }}P\left(G_{t}\geq r\right)+\sum_{r=\tau^{\prime }}^{\infty }P\left(G_{t}\geq r\right)\\ & \leq 1+\frac{6}{\tau }\log\left(\tau +e^{11}\right). \end{array}$$

Therefore, when $N_{t}\left(\tau ,*\right)>L\left(\tau \right)$, it holds that

$$E\left[\frac{1}{p_{i,t}}\right]-1=E\left[G_{t}\right]-1\leq \frac{6}{\tau }\log\left(\tau +e^{11}\right).$$

**Step 3:** Let $A\left(\tau ,*\right)=\{t\in \left\{1,\ldots ,T\right\}:N_{t}\left(\tau ,*\right)\leq L\left(\tau \right)\}$ and $C=e^{11}+9$.

(A19) $$\begin{array}{cc} \; & \sum_{t\in T(\tau )}E\left[\frac{1-p_{i,t}}{p_{i,t}}1\left\{i_{t}=i_{t}^{*},\theta_{t}\left(i\right)<y_{t}\left(i\right)\right\}\right]\\ \; & \leq \left(\sum_{t\in T(\tau )\cap A(\tau ,*)}+\sum_{t\in T(\tau )\setminus A(\tau ,*)}\right)E\left[\frac{1-p_{i,t}}{p_{i,t}}1\left\{i_{t}=i_{t}^{*},\theta_{t}\left(i\right)<y_{t}\left(i\right)\right\}\right]\\ \; & \leq |\left\{t:i_{t}=i_{t}^{*},N_{t}\left(\tau ,*\right)\leq L\left(\tau \right)\right\}|\left(e^{11}+3+3\log\tau \right)+\sum_{t\in T(\tau )\setminus A(\tau ,*)}E\left[\frac{1-p_{i,t}}{p_{i,t}}\right]\\ \; & \leq \frac{T}{\tau }L\left(\tau \right)\left(e^{11}+3+3\log\tau \right)+6\frac{T}{\tau }\log\left(\tau +e^{11}\right)\\ \; & \leq \left(e^{11}+9+3\log\tau \right)\frac{T}{\tau }L\left(\tau \right). \end{array}$$
